# The Modified Budin View: A Reliable and Accessible Screening Tool for Femoral Version Deformities in Joint-Preserving Hip Surgery

**DOI:** 10.1177/03635465261439075

**Published:** 2026-05-05

**Authors:** Alexander F. Heimann, Jason Carrel, Florian Schmaranzer, Vera M. Stetzelberger, Joseph M. Schwab, Matthieu Hanauer, Darius Marti, Jonathan Laurencon, Moritz Tannast

**Affiliations:** † Department of Orthopaedic Surgery and Traumatology, HFR–Cantonal Hospital, University of Fribourg, Fribourg, Switzerland; ‡ Department of Radiology, Balgrist University Hospital, Zurich, Switzerland; § Department of Orthopaedic Surgery and Traumatology, Valais Hospital, Martigny, Switzerland; ‖ Department of Orthopaedic Surgery, Inselspital Bern, University Hospital, University of Bern, Bern, Switzerland; Investigation performed at HFR–Cantonal Hospital, University of Fribourg, Fribourg, Switzerland

**Keywords:** femoral version, modified Budin view, diagnostic imaging, joint-preserving hip surgery

## Abstract

**Background:**

Femoral version deformities are key in the diagnostic evaluation of young patients with hip pain. While computed tomography (CT) and magnetic resonance imaging (MRI) are commonly used for femoral version assessment, their limitations include a lack of standardized reference values, high costs, and accessibility constraints. An accessible and reproducible alternative is needed, particularly in the context of joint-preserving hip surgery.

**Purpose:**

To evaluate the reliability and reproducibility of femoral version measurements on the modified Budin view, to compare these measurements with 4 established cross-sectional imaging methods (CT or MRI), and to determine the diagnostic accuracy of the modified Budin view in identifying abnormal femoral version.

**Study Design:**

Case series; Level of evidence, 4.

**Methods:**

The authors retrospectively analyzed the records of 93 patients (107 hips; mean age, 31 ± 9 years [range, 16-55 years]; 43 female hips [40%]) evaluated for joint-preserving hip surgery at a specialized tertiary center. Femoral version was measured using the modified Budin view and compared to 4 cross-sectional imaging methods (CT and MRI) using Bland-Altman analysis and diagnostic accuracy metrics.

**Results:**

The mean femoral version measured on the modified Budin view was 13°± 7° (95% CI, 12°-14°). The technique demonstrated excellent intra- and interobserver reliability (ICC, 0.99 and 0.98, respectively). Compared to the Reikerås method, the mean difference was 1°± 4° (95% CI, 0°-2°; *P* = .042; Bonferroni-corrected significance level, *P* < .01). The mean differences were 3°± 5° (95% CI, 2°-3°) for the Lee method, 11°± 5° (95% CI, 10°-12°) for the Tomczak method, and 12°± 6° (95% CI, 11°-14°) for the Murphy method (all *P* < .001). Diagnostic accuracy for detecting abnormal femoral version was high, with an area under the curve ranging from 0.88 to 0.98 and a consistently high negative predictive value (91%-100%).

**Conclusion:**

The modified Budin view is a highly reliable and accessible radiographic method for femoral version measurement in young patients undergoing joint-preserving hip surgery. It closely reflects the femoral neck version as measured by the Reikerås method, and demonstrates high diagnostic accuracy for detecting abnormal femoral version, making it a valuable screening tool for femoral version deformities in clinical practice.

Femoral version deformities are increasingly recognized as key contributors to hip pain, instability, femoroacetabular impingement, and the development of osteoarthritis in young patients.^6,11,14,25,30-32^ While computed tomography (CT) remains the gold standard for femoral version measurement,^23,27^ magnetic resonance imaging (MRI) provides a reliable alternative for assessing femoral version in both pediatric and adult populations.^5,7,22^ However, cross-sectional imaging is resource-intensive, costly, and often inaccessible in settings lacking specialized equipment or expertise. It may also be impractical for patients with limited mobility or tolerance for prolonged imaging studies. Furthermore, multiple measurement methods exist, each relying on different anatomic landmarks, leading to femoral version values that can vary by up to 17° in the same patient.^23^ This variability hampers the establishment of standardized reference values and limits interstudy comparability.^6,32^ With no consensus on the optimal measurement technique, institutional preferences vary, further contributing to inconsistencies. These limitations highlight the need for a simple, cost-effective, and widely accessible alternative for femoral version assessment.

In 1957, Budin and Chandler^4^ proposed a single-view radiographic technique for femoral version measurement. More recently, this technique was reintroduced in a modified form for assessing femoral stem version in patients undergoing total hip arthroplasty^12,17^ and those with end-stage hip osteoarthritis.^3^ In these older populations, the modified Budin view has demonstrated excellent intra- and interobserver reliability, as well as high correlation with CT-based measurements. However, its applicability to young patients undergoing evaluation for joint-preserving hip surgery has not been thoroughly investigated to date.

To address these gaps, we investigated a cohort of young patients undergoing evaluation for joint-preserving hip surgery and asked the following: (1) How reliable and reproducible are femoral version measurements on the modified Budin view? (2) How do femoral version measurements on the modified Budin view compare to 4 methods based on cross-sectional imaging (CT or MRI) using Bland-Altman analysis? (3) What is the diagnostic accuracy of the modified Budin view in differentiating between normal and abnormal femoral version (increased or decreased) on cross-sectional imaging?

## Methods

### Study Design and Settings

This was a single-center, retrospective, method comparison and diagnostic accuracy study conducted at the Cantonal Hospital – University of Fribourg, Fribourg, Switzerland. The study protocol was approved by the local ethics committee before initiation of data collection (Project-ID 2023-02199). We performed a retrospective review of the radiographic records of all patients who underwent a modified Budin view for femoral version assessment during scheduled visits to the outpatient clinic of our tertiary center for joint-preserving hip surgery between September 2023 and November 2023.

### Study Participants

**Figure 1.**
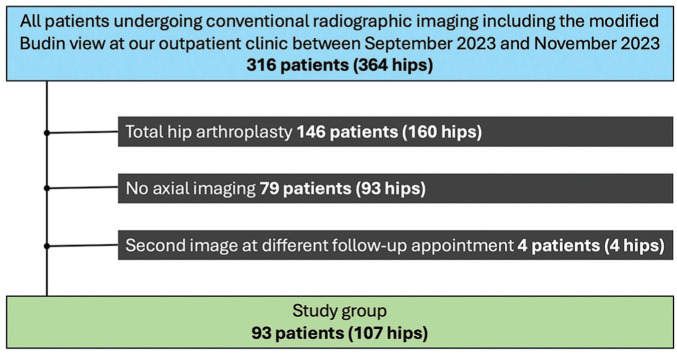
Study flowchart.

A total of 316 patients (364 hips) met the initial inclusion criteria. Exclusion criteria consisted of patients who had undergone total hip arthroplasty (146 patients [160 hips]); patients without cross-sectional imaging (CT or MRI) including the distal femoral condyles, which are required for femoral version calculation (79 patients [93 hips]); and patients with repeated modified Budin views of the same hip at different follow-up appointments (4 patients [4 hips]). After applying these criteria, the final study cohort consisted of 93 patients (107 hips) (Figure 1).

### Patient Characteristics

The mean age of the study group was 31 ± 9 years (range, 16-55 years), there were 43 female hips (40%), and the mean body mass index was 23.6 ± 2.9 kg/m^2^ (range, 19.4-35.5 kg/m^2^) (Table 1).

**Table 1:** Patient Characteristics*^*a*^*

	Value
Hips/patients	107/93
Age, y	31 ± 9 (16-55)
Female sex	43 (40)
Right side	63 (59)
Height, m	1.75 ± 0.09 (1.56-1.94)
Weight, kg	73 ± 13 (51-110)
BMI, kg/m^2^	23.6 ± 2.9 (19.4-35.5)
Previous surgery	67 (63)
Surgical hip dislocation	48 (45)
Periacetabular osteotomy	8 (7)
Hip arthroscopy	7 (7)
Total hip arthroplasty	00 (0)
Other	7 (7)

a Continuous data are presented as mean ± SD (range). Categorical data are presented as n (%). BMI, body mass index.

### Radiographic Evaluation

Conventional radiographs, including anteroposterior pelvis radiographs and axial cross-table views in the supine position, were acquired in all patients using a standardized technique.^28^ Radiographic evaluation of femoroacetabular morphology on plain radiographs included the following measurements (Table 2): lateral center-edge angle,^33^ acetabular index,^30^ extrusion index,^18^ neck-shaft angle,^2^ femoroepiphyseal acetabular roof index,^34^ and radiographic signs of acetabular retroversion (ischial spine sign, posterior wall sign, crossover sign, and retroversion index^8,15,35^). The alpha angle was measured on the axial cross-table views.^20^ Osteoarthritis grading was performed according to the Tönnis classification.^10^

**Table 2:** Radiographic Hip Parameters*^*a*^*

	Value*^*b*^*	95% CI
Lateral center-edge angle, deg	31 ± 7 (15 to 50)	29 to 32
Acetabular index, deg	3 ± 7 (–27 to 26)	2 to 5
Extrusion index, %	14 ± 7 (–4 to 29)	13 to 16
Central acetabular version, deg	19 ± 14 (–1 to 80)	17 to 22
FEAR index, %	−17 ± 12 (–58 to 9)	−20 to −15
Neck-shaft angle, deg	131 ± 6 (111 to 148)	130 to 132
Femoral version, deg	25 ± 10 (–3 to 62)	23 to 27
Alpha angle, deg	44 ± 11 (30 to 96)	42 to 46
Crossover sign	39 (36)	27 to 46
Ischial spine sign	57 (53)	43 to 63
Posterior wall sign	82 (77)	67 to 84
Retroversion index	14 ± 21 (0 to 100)	10 to 18
Tönnis grade >0	44 (41)	32 to 51

a FEAR, femoroepiphyseal acetabular roof.

b Data are presented as n (%) or mean ± SD (range).

### Cross-sectional Imaging

All patients underwent cross-sectional imaging of the pelvis and distal femoral condyles, including CT in 32 patients (37 hips), magnetic resonance arthrography (MRA) in 27 patients (30 hips), or both CT and MRA in 34 patients (40 hips), allowing calculation of femoral version. In the latter group, femoral version was measured on either CT or MRA images, with patients randomly assigned to 1 modality: half were assessed using CT, and the other half using MRA. Overall, femoral version was measured on CT in 49 patients (57 hips [53%]) and on MRA in 44 patients (50 hips [47%]). Measurements from both imaging modalities were analyzed together, as prior studies have shown that MR- and CT-based femoral version measurements demonstrate high agreement and comparable reliability and reproducibility, with intraclass correlation coefficient (ICC) values ranging from 0.97 (95% CI, 0.95-0.98) to 0.99 (95% CI, 0.98-0.99) for both modalities, with intermodal differences falling within the variation of the corresponding ICC.^22^

Noncontrast CT imaging was performed using a dual-source CT scanner (Revolution CT; GE HealthCare) following our institutional protocol. Patients were positioned supine with feet fixed in internal rotation and knees fully extended. The field of view included the pelvis and distal femoral condyles, with a slice thickness of 0.625 mm.

MRA was performed as direct imaging at 3 T (SIGNA Premier; GE HealthCare) using a large flexible body coil for the pelvis region and a spine matrix coil for the knee region. For acquisition of images of the pelvis and the distal femoral condyles, no traction was applied, and patient positioning was identical to that described for the CT studies above, with a slice thickness of 3 mm. Femoral version measurements were carried out on fast T1-weighted gradient-echo sequences (liver acquisition with volume acceleration flexibility), with additional multiplanar proton density–weighted images acquired for evaluation of intra-articular pathologies.

### Measurement of Femoral Version

**Figure 2.**
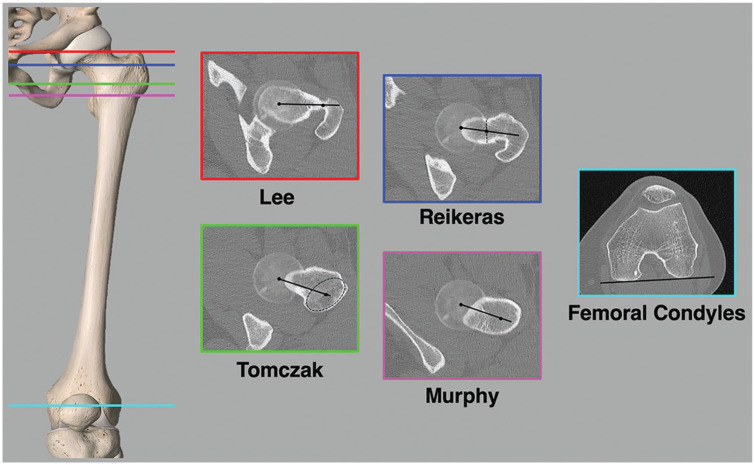
Anatomic relationship between the 4 cross-sectional femoral version measurement methods, with corresponding axial images showing the respective proximal reference lines. The distal reference line, connecting the apices of the lateral and medial posterior femoral condyles, was consistent across all methods.

Femoral version was measured in all patients using true axial images, following 4 established gold-standard measurement methods (Figure 2). A common distal reference axis was defined as the line connecting the apices of the posterior lateral and medial femoral condyles, while the proximal reference axis varied depending on the anatomic landmarks specific to each method.

The most proximal measurements followed the method of Lee et al,^13^ identifying the axis at the first image where the femoral head center (FHC), femoral neck, and most cephalic junction of the greater trochanter could be connected. Slightly more distal measurements utilized the Reikerås^21^ method, where the FHC is connected with the femoral neck axis on the cross-sectional image, where the anterior and posterior neck cortices are parallel. The method described by Tomczak et al^29^ defined the proximal reference axis further distally by connecting the FHC with the midpoint of the greater trochanter at the base of the femoral neck. Finally, the most distal measurements were performed according to Murphy et al,^19^ at the level of the superior end of the lesser trochanter. One observer (A.F.H.), an orthopaedic resident with 4 years of experience in diagnostic hip imaging, performed femoral version measurements using all 4 methods in each patient.

### Modified Budin View

**Figure 3.**
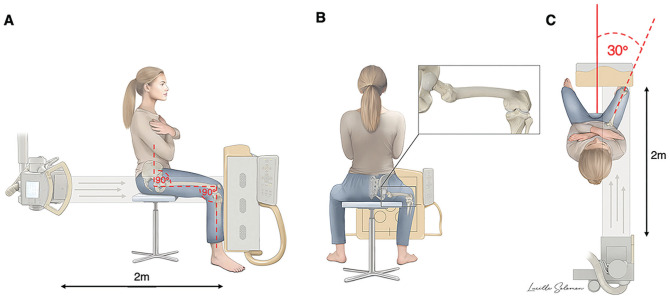
Acquisition technique of the modified Budin view for single-view radiographic measurement of femoral version. (A) Lateral view showing a female patient in the seated position with the hip and knee flexed at 90°. (B) Posterior and (C) superior views showing an additional 30° of hip abduction and a schematic representation of the obtained modified Budin view (inset).

A modified Budin view was obtained in all patients using posteroanterior radiographs taken with the patient in the seated position, the hip and knee flexed to 90°, and the hip abducted to 30°. The film-focus distance was set at 2 m, and the x-ray beam was centered on the hip joint (Figure 3).

**Figure 4.**
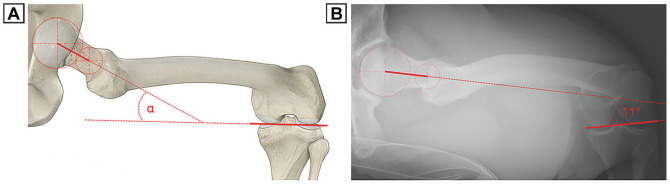
(A) Illustration of the femoral version measurement methodology on the modified Budin view. Femoral version is measured as the angle α between the proximal reference line (connecting the femoral head center and the center of the femoral neck) and the distal reference line (connecting the apices of the lateral and medial posterior femoral condyles). (B) Modified Budin view showing a femoral version measurement.

For the single-view radiographic measurements of femoral version, the proximal reference axis was defined as the line connecting the FHC with the center of the femoral neck, while the distal reference axis was defined as the line connecting the apices of the posterior lateral and medial femoral condyles. Femoral version was measured as the angle between these 2 reference lines (Figure 4).

### Reliability and Reproducibility of the Modified Budin View

To assess the reliability of femoral version measurements on the modified Budin view, the primary observer (A.F.H.), an orthopaedic resident with 4 years of experience in diagnostic hip imaging, measured all hips twice, with a 4-week interval between the 2 measurement sessions. The order of measurements in the second session was randomized, and the observer was blinded to the initial results.

Reproducibility was evaluated by comparing the measurements taken by a second independent observer (V.M.S.), an orthopaedic resident with 6 years of experience in diagnostic hip imaging, to those from the first observer. Both observers were blinded to each other's results to ensure objective assessment. The same measurement protocol was applied consistently to all hips.

### Validation Against Cross-sectional Measurement Methods

To evaluate intermodal differences in femoral version measurements, measurements obtained using the modified Budin view were compared to the 4 cross-sectional methods using Bland-Altman analysis. This allowed for insights into whether and how the methods systematically differed and whether the differences were influenced by the magnitude of the measurements.

### Diagnostic Accuracy of the Modified Budin View

To evaluate the diagnostic accuracy of the modified Budin view, normal femoral version was defined for each cross-sectional method as the mean ± 1 SD of femoral version reported in asymptomatic adults. Depending on the method used, normal values of femoral version varied, ranging from 9°± 10° when measured according to the Reikerås method to 22°± 8° when measured according to Murphy et al.^9,14,21,26^

Diagnostic thresholds, which represent specific cutoff values used to differentiate between normal and abnormal measurements, were subsequently established to evaluate the modified Budin view's ability to identify deviations in femoral version. These thresholds help determine the point at which a measurement is most likely to indicate a clinically significant abnormality. Ideal diagnostic thresholds for femoral version on the modified Budin view were subsequently determined, and corresponding diagnostic accuracy metrics were calculated.

### Selection Bias

To minimize selection bias, we included a consecutive series of patients in our study, ensuring that all patients who met the inclusion criteria had an equal chance of being selected. To enhance comparability and generalizability, we used a previously described acquisition method for the modified Budin view.^12^ During the evaluation of measurement reliability, the order of measurements was randomized during the second session, and the observer was blinded to the initial results to minimize recall bias. Additionally, interrater reliability was assessed to evaluate the consistency between 2 observers.

### Statistical Analysis

Data analyses were performed using MedCalc (Version 22.023; MedCalc Software Ltd). Power analysis was conducted performed G*Power (Version 3.1.9.6; Heinrich Heine Universität, Düsseldorf, Germany). An a priori power analysis for the difference between 2 dependent means was conducted. Effect size estimation was based on a previously reported mean intermodal difference of the modified Budin view and a CT-based measurement in osteoarthritic hips^12^ and calculated as 0.28. With an alpha error of .05 and a power of 80%, the required sample size was determined to be 103 hips.

Intrarater reliability and interrater reproducibility were assessed using the ICC,^16^ interpreted as follows: poor (<0.4), fair (0.41-0.60), good (0.61-0.80), excellent (0.81-0.90), and almost perfect (>0.90). Subsequent analyses were based on the primary observer's measurements after confirmation of high reproducibility and reliability.

Femoral version measurements were tested for normality using the Kolmogorov-Smirnov test. Pairwise comparisons between the modified Budin view and each cross-sectional measurement method were performed using dependent-samples *t* tests with Bonferroni correction for multiple testing, yielding a threshold of *P* < .01 to determine statistical significance.

Agreement between methods was evaluated using Bland-Altman analysis. Differences between methods were plotted against the mean, with limits of agreement defined as ±1.96 SD. Bias was quantified as the mean difference between the modified Budin view and the corresponding cross-sectional method, reported with 95% confidence intervals. Proportional bias, which suggests that the magnitude of the measurement error depends on the size of the measurement itself, was assessed using least squares regression, with a nonzero slope indicating its presence. Heteroscedasticity, or variability in measurement differences that changes depending on the size of the measurement, was evaluated by analyzing the 95% confidence interval of the regression line of differences.

Receiver operating characteristic curves were constructed to determine the diagnostic thresholds of the modified Budin view for identifying hips with increased or decreased femoral version as defined by each cross-sectional method. Diagnostic metrics, including the area under the curve (AUC), sensitivity, specificity, positive predictive value (PPV), and negative predictive value (NPV), were calculated.

## Results

### Reliability and Reproducibility of Femoral Version Measurements on the Modified Budin View

The intrarater reliability ICC was 0.99 (95% CI, 0.98-0.99), and interrater reproducibility ICC was 0.98 (95% CI, 0.97-0.99) (Table 3).

**Table 3:** Femoral Version Across Measurement Methods: Descriptive Statistics and Reliability Metrics*^*a*^*

Method	Imaging Modality	Femoral Version, deg*^*b*^*	Range	Intraobserver ICC*^*c*^*	Interobserver ICC*^*c*^*
Lee	CT/MRA	10 ± 10 (8-12)	−17 to 38	0.98 (0.96-0.99)^21^	0.98 (0.95-0.99)^21^
Reikerås	CT/MRA	12 ± 9 (10-14)	−8 to 41	0.98 (0.96-0.99)^21^	0.98 (0.96-0.99)^21^
Modified Budin	Conventional radiograph	13 ± 7 (12-14)	−5 to 31	0.99 (0.98-0.99)	0.98 (0.97-0.99)
Tomczak	CT/MRA	24 ± 9 (23-26)	00 to 52	0.99 (0.98-0.99)^21^	0.98 (0.97-0.99)^21^
Murphy	CT/MRA	25 ± 11 (23-27)	−3 to 62	0.99 (0.98-0.99)^21^	0.98 (0.98-0.99)^21^

a CT, computed tomography; ICC, intraclass correlation coefficient; MRA, magnetic resonance arthrography.

b Data are presented as mean ± SD (95% CI).

c Data are presented as correlation (95% CI).

### Comparison of Femoral Version Measurements: Modified Budin View Versus Cross-sectional Imaging

The mean femoral version measured on the modified Budin view was 13°± 7° (95% CI, 12°-14°). On cross-sectional imaging, femoral version increased with more distal definitions of the proximal anatomic reference line and differed between methods (all *P* < .001) (Table 4).

**Table 4:** Pairwise Comparison of Differences Between the Measurement Methods for Femoral Version*^*a*^*

Method	Modified Budin	Lee	Reikerås	Tomczak	Murphy
Modified Budin	—	3 ± 5 (2-3)	1 ± 4 (0-2)	11 ± 5 (10-12)	12 ± 6 (11-14)
Lee	<.001	—	2 ± 3 (1-3)	14 ± 3 (14-15)	15 ± 5 (14-16)
Reikerås	.042	<.001	—	12 ± 4 (11-13)	13 ± 5 (12-14)
Tomczak	<.001	<.001	<.001	—	1 ± 3 (0-2)
Murphy	<.001	<.001	<.001	<.001	—

a Data are presented as mean difference (in degrees) of femoral version ± SD (95% CI) or *P* value. The *P* values represent the level of significance for each comparison; dashes represent the cells for which no pairwise comparison was possible.

The mean femoral versions were 10°± 10° (Lee), 12°± 9° (Reikerås), 24°± 9° (Tomczak), and 25°± 11° (Murphy).

**Figure 5.**
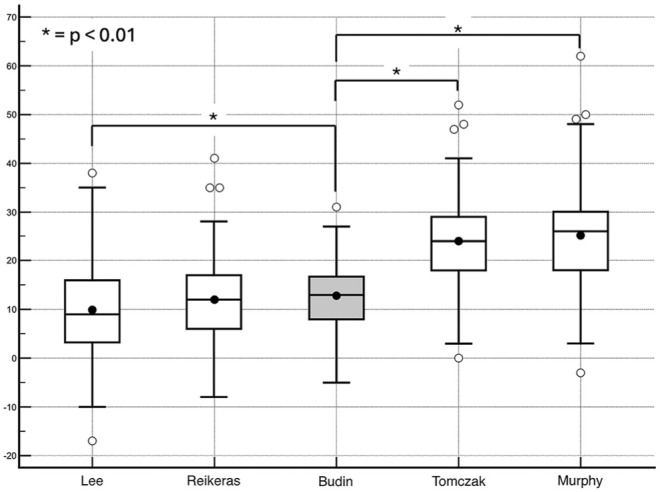
Box plots illustrating femoral version measurements obtained through 4 cross-sectional methods (white box plots: Lee, Reikerås, Tomczak, and Murphy) compared to the single-view radiographic measurement on the modified Budin view. A Bonferroni correction for multiple comparisons was applied, establishing a significance threshold of *P* < .01. Significant differences were found between the modified Budin view and the cross-sectional methods by Lee, Tomczak, and Murphy (all *P < .01), whereas no significant difference was observed between the modified Budin view and the Reikerås method (*P* = .042). Detailed results can be found in Tables 3 and 4.

The mean values from the modified Budin view did not differ from those of Reikerås, but did differ from those of the other 3 methods (Figure 5).

**Figure 6.**
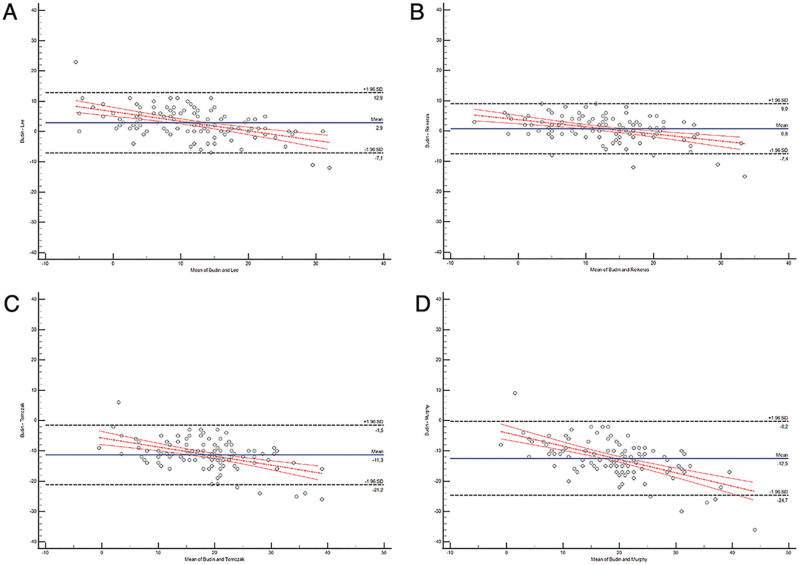
Bland-Altman analysis to assess systematic bias between the modified Budin view and the 4 cross-sectional imaging-based femoral version measurement methods: (A) Budin versus Lee, (B) Budin versus Reikerås, (C) Budin versus Tomczak, and (D) Budin versus Murphy. Limits of agreement are shown as ± 1.96 SD (–7 to 13 for Lee, −7 to 9 for Reikerås, −21 to −1 for Tomczak, and −25 to 0 for Murphy). Mean values of bias are represented by the solid blue line. The regression lines of differences, with corresponding 95% confidence intervals, are shown in red. The respective slopes of the regression lines are −0.32 for Lee, −0.24 for Reikerås, −0.30 for Tomczak, and −0.44 Murphy.

Mean differences between the Budin view and cross-sectional methods were 1°± 4° (Reikerås), 3°± 5° (Lee), 11°± 5° (Tomczak), and 12°± 6° (Murphy) (Table 4, Figure 6).

Bland-Altman analysis revealed varying degrees of proportional bias and heteroscedasticity between the modified Budin view and all cross-sectional methods (Figure 6), resulting in measurement discrepancies of up to 18° between the modified Budin view and the cross-sectional methods in patients with abnormal femoral version (Table 5).

**Table 5:** Comparison of Mean Differences Between the Cross-sectional Methods and the Modified Budin View in Patients With Abnormally High/Low Femoral Version

Cross-sectional Method	Threshold, deg*^*a*^*	No. of Hips	Cross-sectional Imaging, deg	Modified Budin View, deg	Mean Difference, deg	*P* Value*^*b*^*
Lee						
Increased femoral version	>19	15	26 ± 6 (23 to 28)	24 ± 4 (22 to 25)	−2 ± 5 (−4 to 0)	.107
Decreased femoral version	<–1	9	−7 ± 4 (−10 to −4)	2 ± 4 (–1 to 4)	9 ± 6 (5 to 13)	**<.001**
Reikerås						
Increased femoral version	>22	11	29 ± 6 (25 to 32)	23 ± 6 (20 to 27)	−5 ± 6 (−9 to −2)	**.016**
Decreased femoral version	<4	18	00 ± 3 (−2 to 1)	3 ± 4 (1 to 5)	4 ± 3 (2 to 5)	**<.001**
Tomczak						
Increased femoral version	>29	26	36 ± 6 (33 to 38)	20 ± 6 (18 to 22)	−15 ± 5 (−17 to −14)	**<.001**
Decreased femoral version	<11	7	6 ± 4 (3 to 9)	1 ± 4 (–2 to 4)	−5 ± 6 (−9 to −1)	.059
Murphy						
Increased femoral version	>30	25	39 ± 7 (36 to 42)	20 ± 6 (18 to 23)	−18 ± 6 (−21 to −16)	**<.001**
Decreased femoral version	<14	14	9 ± 5 (6 to 11)	3 ± 4 (1 to 5)	−6 ± 5 (−9 to −3)	**.009**

a According to reported normal values in asymptomatic patients.

b Boldface type indicates statistical significance.

### Diagnostic Accuracy of the Modified Budin View for Identifying Normal and Abnormal Femoral Version

The modified Budin view demonstrated excellent discriminative ability for distinguishing between normal and abnormal femoral version, with high NPVs across all measurement methods (Table 6).

**Table 6:** Diagnostic Thresholds for the Modified Budin View to Differentiate Between Increased and Decreased Femoral Version*^*a*^*

Method	Threshold on Modified Budin View, deg	No. of Hips	Sensitivity, %	Specificity, %	AUC	95% CI	PPV, %	NPV, %
Lee								
Increased femoral version (>19°)	>19	15	93	96	0.98	0.93-1.00	78	99
Decreased femoral version (<–1°)	≤8	9	100	79	0.95	0.89-0.98	30	100
Reikerås								
Increased femoral version (>22°)	>22	11	82	98	0.92	0.86-0.97	82	98
Decreased femoral version (<4°)	≤8	18	94	85	0.96	0.90-0.99	57	99
Tomczak								
Increased femoral version (>29°)	>18	26	69	95	0.88	0.80-0.93	82	91
Decreased femoral version (<11°)	≤6	7	100	85	0.96	0.90-0.99	32	100
Murphy								
Increased femoral version (>30°)	>19	25	68	99	0.88	0.80-0.93	94	91
Decreased femoral version (<14°)	≤6	14	86	89	0.95	0.89-0.98	55	98

a AUC, area under the curve; NPV, negative predictive value; PPV, positive predictive value.

For detecting decreased femoral version, thresholds on the modified Budin view were ≤8° for the more proximal methods (Lee and Reikerås) and ≤6° for the more distal methods (Tomczak and Murphy). These thresholds yielded sensitivities of 86% to 100% and specificities of 79% to 89%, with AUCs ranging from 0.95 (95% CI, 0.89-0.98) to 0.96 (95% CI, 0.90-0.99). PPVs ranged from 30% to 57%, while NPVs were 98% to 100%.

For increased femoral version, thresholds on the modified Budin view were >19° (Lee), >22° (Reikerås), >18° (Tomczak), and >19° (Murphy). These thresholds yielded sensitivities of 68% to 93% and specificities of 95% to 99%, with AUCs ranging from 0.88 (95% CI, 0.80-0.93) to 0.98 (95% CI, 0.93-1.00). PPVs ranged from 78% to 94%, while NPVs were 91% to 99%.

## Discussion

Femoral version deformities play a key role in the diagnostic evaluation of young patients with hip pain. CT and MRI provide reliable measurements^5,22,23,27^ but are limited by cost and accessibility. The variety of measurement methods and lack of standardized reference values hinder interstudy comparability. The terms “femoral version” and “femoral torsion” are often used interchangeably in the literature, and no universally accepted distinction currently exists. Recent anatomic work has proposed a more granular definition, in which femoral version refers to the anterior orientation of the femoral neck, femoral torsion describes rotation of the femoral shaft, and total femoral version encompasses the combined contribution of both components.^1^ Accordingly, rather than attributing the modified Budin view to either torsion or version alone, it is important to recognize that this radiographic view quantifies the angular orientation of the femoral neck relative to the distal femur, without isolating the specific anatomic site of rotational contribution.

In the context of value-based health care and the increasing demand for joint-preserving hip procedures, a cost-effective, accessible screening tool for femoral version assessment is highly warranted. In this study, we therefore evaluated the clinical utility of the modified Budin view, a single-view radiographic method, as a reliable and practical alternative for measuring femoral version in young patients undergoing evaluation for joint-preserving hip surgery. Our findings demonstrate excellent intra- and interobserver reliability, strong concordance with the Reikerås method, and high diagnostic accuracy for detecting abnormal femoral version across all 4 cross-sectional methods. Notably, the modified Budin view provides a high NPV, supporting its potential as a reliable and widely available screening tool in clinical practice.

Mean femoral version measured on the modified Budin view was 13°± 7° (95% CI, 12°-14°), aligning with previously reported values of femoral version in older patients with end-stage hip osteoarthritis (13°± 9°).^3^ The method demonstrated excellent intraobserver reliability (ICC, 0.99; 95% CI, 0.98-0.99) and interobserver reproducibility (ICC, 0.98; 95% CI, 0.97-0.99). These findings likely reflect the use of standardized measurement protocols and a consistent radiographic setup and are consistent with previous studies assessing this technique in older patients, in which ICCs have ranged from 0.89 (95% CI, 0.84-0.91) to 0.99 (95% CI, 0.99-0.99).^3,12,17^

Additionally, the modified Budin view's measurement reliability is on par with cross-sectional imaging methods, where ICC values for CT- or MRI-based measurements range from 0.90 (95% CI, 0.86-0.94) to 0.99 (95% CI, 0.98-0.99).^7,14,22,23^ This underscores the technique's precision and repeatability, further supporting its clinical utility.

A key advantage of this single-view radiographic method is that all required reference lines are captured on a single image. In contrast, CT and MRI require image superposition to allow for femoral version measurements, introducing potential selection bias on the chosen reference slices. In MRI-based assessments, longer acquisition times may theoretically allow for patient movement, potentially resulting in differing proximal and distal femoral positions. However, when standardized protocols with fixed foot positioning and rapid MRI sequences (eg, VIBE-Dixon) are used, the likelihood of relevant intersequence leg position changes is low.

Femoral version measurements on the modified Budin view closely matched those obtained via the Reikerås method (mean difference, 1°± 4°, 95% CI, 0°-2°; *P* = .042) (Table 4, Figure 5), suggesting that reported normal values (13°± 7° in asymptomatic adults)^21^ may also be applicable to the modified Budin view.

This finding may be explained by the shared anatomic reference lines. While all methods use the same distal reference line, only the modified Budin view and the Reikerås method share the same proximal reference line (Figures 2 and 4).

Consistent with our findings, Lee et al^12^ reported no significant differences between the modified Budin view and CT-based femoral version measurements in patients with total hip arthroplasty. In contrast, Boissonneault et al^3^ found a statistically significant difference between femoral version measurements on the modified Budin view and the Reikerås method. However, the observed magnitude of difference (1°) was similar to that in our study, and after removing 3 outliers, no significant difference remained.^3^

In contrast, significant differences were observed between the modified Budin view and the 3 other cross-sectional methods (Table 4, Figure 5). The more proximal measurement method by Lee et al yielded lower differences in femoral version values (mean difference, 3°± 5°; 95% CI, 2°-3°), while the more distal methods by Tomczak and Murphy showed greater discrepancies (Tomczak: 11°± 5° [95% CI, 10°-12°]; Murphy: 12°± 6° [95% CI, 11°-14°]).

Bland-Altman analysis demonstrated strong agreement between the modified Budin view and the Reikerås method, with a mean bias of 0.8° (95% CI, 0.0° to 1.6°). This finding is consistent with the results of Boissonneault et al,^3^ who reported a fixed bias of −0.8° (95% CI, −1.4° to −0.2°) in older patients.

However, proportional bias was observed across all cross-sectional methods, with the modified Budin view overestimating femoral version at lower values and underestimating it at higher values (Table 5, Figure 6). This systematic trend indicates that measurement accuracy varies depending on the degree of femoral version deformity. In addition, heteroscedasticity—increased variability between methods at the extremes—was observed for both low and high femoral version. This pattern has been previously described and precludes the use of simple mean adjustments to convert values between the modified Budin view and cross-sectional methods.^23^

By providing a single-view measurement with defined reference ranges, the modified Budin view could offer a simplified and more accessible approach for femoral version assessment in daily clinical practice.

The modified Budin view demonstrated excellent diagnostic performance. While PPV varied widely (30%-94%), NPV remained consistently high (91%-100%), reinforcing its utility as a screening tool. This high NPV suggests that the modified Budin view is particularly useful for ruling out abnormal femoral version when radiographic findings fall within the normal range. Specifically, femoral version between 9° and 18° on the modified Budin view correctly identified patients with normal femoral version according to all 4 measurement methods (Table 6). This consistency across methods supports the classification of femoral version in the 9° to 18° range on the modified Budin view as normal.

However, the PPV for abnormally low femoral version was lower and varied across cross-sectional methods. Accordingly, we recommend using the Budin view primarily as a screening tool. Abnormal values, particularly low version, should be confirmed with CT or MRI before definitive surgical planning. Strict adherence to standardized patient positioning and rater training may further improve measurement accuracy and reliability in clinical practice. Although advanced imaging remains essential for detailed preoperative planning, the modified Budin view as a screening tool for femoral version could allow the surgeon to order more targeted advanced imaging protocols, allowing for more efficient resource allocation in clinical practice. In addition, patients often present with prior cross-sectional imaging. While these scans may suffice for assessing bony and soft tissue morphology at the level of the hip, they often exclude the distal femoral condyles, necessitating repeat imaging for femoral version measurement. By identifying patients with normal femoral version, the modified Budin view may help reduce unnecessary repeat imaging.

Beyond its diagnostic accuracy, the modified Budin view holds clinical value in early detection of femoral version deformities that contribute to pain, instability, and progressive joint degeneration. Early recognition may facilitate timely intervention, potentially delaying or preventing the onset of advanced osteoarthritis. Moreover, its ease of implementation makes it a practical addition to routine radiographic assessments in outpatient and resource-limited settings.

**Figure 7.**
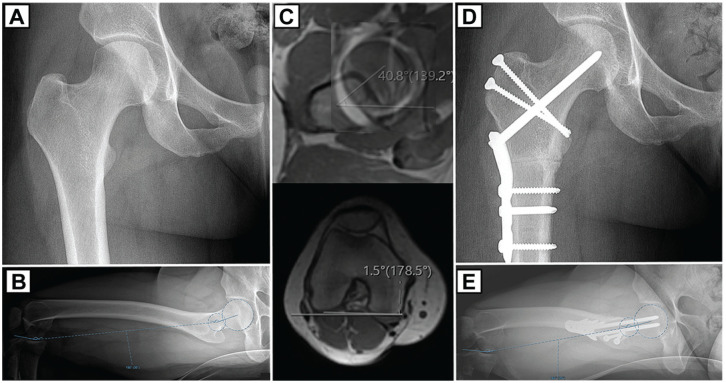
(A) Anteroposterior pelvis radiograph obtained in a 25-year-old female patient with right hip pain. (B) The modified Budin view demonstrates a femoral anteversion of 30°, suggestive of increased femoral version. (C) Magnetic resonance imaging confirmed an increased femoral version of 43° according to Murphy. (D) Postoperative anteroposterior pelvis radiograph obtained after subtrochanteric derotation osteotomy via surgical hip dislocation. (E) The postoperative modified Budin view enables quantification of the correction, with postoperative femoral version measuring 20°.

Future research should focus on validating the modified Budin view in larger, more diverse populations and exploring its utility in specific clinical scenarios, such as postsurgical follow-up or the evaluation of developmental hip disorders (Figure 7).

Additionally, comparing the modified Budin view with emerging imaging technologies, such as low-dose CT or 3-dimensional (3D) MRI reconstructions, may also provide further insights into optimizing femoral version assessment. Investigating its role in preoperative planning and postsurgical monitoring could further define its place in clinical workflows.

This study has several limitations. First, its retrospective design may introduce selection bias, as the study cohort consisted of patients undergoing evaluation at a specialized tertiary center for joint-preserving hip surgery. Second, while the modified Budin view demonstrated strong agreement with the Reikerås method, it is not a direct replacement for CT or MRI in cases in which detailed anatomic visualization is required. This may be especially true in cases with excessively high femoral version and a valgus deformity in which measurement based on more proximal landmarks, including the Budin view, cannot capture the full extent of the rotational deformity as shown with 3D CT–based simulation.^24^ Specifically, its ability to assess femoral version in patients with complex anatomic variations, such as the sequelae of pediatric hip diseases, remains untested.

Third, technical factors, including patient positioning, radiographic beam alignment, and operator expertise, may affect measurement consistency. Subtle variations in pelvic tilt, limb rotation, or soft tissue interference could impact measurement consistency, particularly in less experienced hands. Training and standardization will be essential for consistent application. Nonetheless, the standardized imaging protocol used in this study allowed for consistent implementation without technical difficulties, underlining that the implementation of the modified Budin view into routine clinical practice should be feasible in most settings.

## Conclusion

The modified Budin view is a highly reliable and accessible single-view radiographic method for assessing femoral version in young patients undergoing evaluation for joint-preserving hip surgery. Its strong agreement with the Reikerås method and high diagnostic accuracy for detecting abnormal femoral version across all cross-sectional methods in this study support its role as a valuable screening tool for femoral version deformities in clinical practice.

By streamlining diagnostic workflows and reducing reliance on resource-intensive imaging, the modified Budin view can enhance the management of femoral version deformities. Its ease of implementation makes it particularly useful in outpatient settings and resource-limited environments, facilitating early detection and timely intervention in young patients with hip pathology.
